# Mechanistic Basis of Antimicrobial Actions of Silver Nanoparticles

**DOI:** 10.3389/fmicb.2016.01831

**Published:** 2016-11-16

**Authors:** Tikam Chand Dakal, Anu Kumar, Rita S. Majumdar, Vinod Yadav

**Affiliations:** ^1^Department of Bio Sciences, Manipal University JaipurJaipur, India; ^2^Department of Biotechnology, School of Engineering and Technology, Sharda UniversityGreater Noida, India; ^3^Department of Microbiology, Central University of HaryanaMahendragarh, India

**Keywords:** silver nanoparticles, multidrug resistance, antimicrobial activity, physico-chemical property, cytotoxicity, genotoxicity, inflammatory response

## Abstract

Multidrug resistance of the pathogenic microorganisms to the antimicrobial drugs has become a major impediment toward successful diagnosis and management of infectious diseases. Recent advancements in nanotechnology-based medicines have opened new horizons for combating multidrug resistance in microorganisms. In particular, the use of silver nanoparticles (AgNPs) as a potent antibacterial agent has received much attention. The most critical physico-chemical parameters that affect the antimicrobial potential of AgNPs include size, shape, surface charge, concentration and colloidal state. AgNPs exhibits their antimicrobial potential through multifaceted mechanisms. AgNPs adhesion to microbial cells, penetration inside the cells, ROS and free radical generation, and modulation of microbial signal transduction pathways have been recognized as the most prominent modes of antimicrobial action. On the other side, AgNPs exposure to human cells induces cytotoxicity, genotoxicity, and inflammatory response in human cells in a cell-type dependent manner. This has raised concerns regarding use of AgNPs in therapeutics and drug delivery. We have summarized the emerging endeavors that address current challenges in relation to safe use of AgNPs in therapeutics and drug delivery platforms. Based on research done so far, we believe that AgNPs can be engineered so as to increase their efficacy, stability, specificity, biosafety and biocompatibility. In this regard, three perspectives research directions have been suggested that include (1) synthesizing AgNPs with controlled physico-chemical properties, (2) examining microbial development of resistance toward AgNPs, and (3) ascertaining the susceptibility of cytoxicity, genotoxicity, and inflammatory response to human cells upon AgNPs exposure.

## Introduction

Unresponsiveness of microbes to lethal doses of structurally diverse classes of drugs with different mechanisms of cytotoxic action is generally referred to as multidrug resistance (MDR). Multidrug resistance of the pathogenic microorganisms to the antimicrobial drugs has become a prime concern toward successful diagnosis and treatment of pathogenic diseases of bacterial and fungal origin (Desselberger, 2000). This has led to emergence and re-emergence of infectious diseases. Indeed, exposure of antimicrobials and antibiotics to bacteria are the opportunities for microbes to become less susceptible toward them mainly by altering the cell structure and cellular metabolism. In this way microbes either destroy the antimicrobials and antibiotics or become unresponsive toward them in future exposures (Desselberger, 2000; Rai et al., 2012). Four mechanisms have been recognized that account for antibiotic resistance in bacteria: (a) alteration of microbial drug target proteins, (b) enzymatic degradation or inactivation of drug, (c) decreased membrane permeability, and (d) increased efflux of drug (Kumar et al., 2013). Among all, the extrusion of antimicrobial drug by the multidrug efflux pumps contributes maximally for MDR among pathogenic strains (Li et al., 1997; Levy, 2002). Although, excessive and irrational use of antibiotics is major factors in development of resistance, the acquisition and dissemination of drug-resistance genes and resistant bacteria have significantly contributed to drug resistance (Davies, 1997; Levy, 2002). Acquisition of drug-resistance generally occurs through genetic mutations, alterations in genetic material or gaining of foreign genetic material (Levy, 2002; Yoneyama and Katsumata, 2006). Dissemination of drug-resistance determinants occurs within genome via transposons or from one microorganism to another by a number of genetic ways, for instance, through transfer of extra-chromosomal element between Gram-positive and Gram-negative bacteria (Levy, 2002). Confronted by the increasing doses of antibiotic drugs over many years, pathogens become drug-resistant and respond to antibiotics by generating progenies that are no more susceptible to antimicrobials therapy (Levy, 2002; Porras-Gomez and Vega-Baudrit, 2012).

Nowadays, non-traditional antimicrobial agents to overcome MDR are increasingly gaining importance. Recently, development of novel, efficient nanotechnological-based antimicrobial agents against multidrug-resistant bacteria is among one of the priority areas in biomedical research (Rai et al., 2012). Silver nanoparticles (AgNPs) display a broad spectrum of antibacterial and antifungal activities (Morones et al., 2005; Kim et al., 2007; Panacek et al., 2009; Namasivayam et al., 2011). Moreover, the advantage of using nanosilver is that it is comparatively less reactive than silver ions, and therefore, is well suited for its use in clinical and therapeutic applications (Kim et al., 2005; Chen and Schluesener, 2008). The antimicrobial activity of AgNPs has been tested against both, MDR and non-MDR strains of bacteria (Feng et al., 2000; Morones et al., 2005; Ayala-Nunez et al., 2009; Humberto et al., 2010; Ansari et al., 2011). In this review, we have presented a comprehensive overview of AgNPs-induced cellular response in bacteria and human cells. AgNPs induce, influence, and modulate diverse range cellular, biochemical, metabolic and inflammatory processes that account for multifaceted antimicrobial activity of AgNPs for tackling multidrug resistance in bacteria. Additionally, some other aspects of AgNPs-based medicines including, physico-chemical properties of AgNPs; cytotoxic, genotoxic and inflammatory response of AgNPs to human cells; and application of AgNPs in therapeutics and targeted drug delivery have also been reviewed.

## MDR and non-MDR strains: bactericidal effect of AgNPs

MDR bacterial strains and infections caused by them are considered as the prime reason for increased mortality rate, morbidity rate and treatment cost in developing countries (Walker et al., 2007; Salem et al., 2015). A number of Gram-positive and Gram-negative bacterial pathogens are known to cause severe medical and clinical complications such as diarrhea, urinary tract disorders, pneumonia, neonatal meningitis etc. (Walker et al., 2007; Salem et al., 2015). Infectious Gram-positive bacteria include *Actinomyces, Bacillus, Clostridium, Corynebacterium, Enterococcus, Listeria, Mycobacterium, Nocardia, Staphylococcus, Streptococcus*, and *Streptomyces*. Among them antibiotic-resistant bacteria are penicillin-resistant *Streptococcus pneumonia*, macrolides resistant *Streptococcus pyogenes*, vancomycin-resistant *Enterococcus faecium* (VREF), methicillin- and vancomycin-resistant *Staphylococcus aureus* (MRSA and VRSA), and multidrug-resistant *Listeria* and *Corynebacterium*. The Gram-negative bacteria include members of the genera *Acinetobacter, Escherichia, Klebsiella, Neisseria, Pseudomonas, Salmonella, Shigella* and *Vibrio*. Among Gram-negative bacteria, *Vibrio cholerae* and enterotoxic *Escherichia coli* (ETEC) are regarded as the two most pathogenic and dominant bacteria that cause severe secretory diarrhea, which significant account for high mortality and morbidity (Salem et al., 2015). Among Gram-negative microbial pathogens some are opportunistic microorganisms, such as *Acinetobacter baumanii, Klebsiella pneumonia*, and *Pseudomonas aeruginosa* that are intrinsically resistant to multiple drugs and infect mainly immune-compromised patients (Levy, 2002). Besides opportunistic pathogens, the strains of *Salmonella typii* have also showed high frequency of drug-resistance and have become resistance to ampicillin, chloroamphenicol, fluoroquinolones, and some other drugs (Levy, 2002). Table 1 contains a list of most common drug-resistant, pathogenic bacterial strains along with the corresponding antibiotics to which the strains have developed resistance.

**Table 1:** **Multidrug-resistant in bacterial strains**.

**Bacterial strains**	**Resistant to**
**GRAM-POSITIVE**	
*Bacillus subtilis*	Chloramphenicol
	Erythromycin Lincomycin
	Penicillin Streptomycin
	Tetracycline
*Corynebacterium diphtheriae*	β-lactam antibiotics Chloramphenicol
	Tetracycline
	Trimethoprim
	Sulfamethoxazole
*Enterococcus faecium*	Vancomycin
	Gentomicin
*Listeria monocytogenes*	Erythromycin
	Gentomicin
	Kanamycin
	Rifampin
	Streptomycin
	Sulfamethoxazole
	Tetracycline
*Staphylococcus aureus*	Methicillin
	Vancomycin
*Streptococcus pneumonia*	Penicillin
	Erythromycin
*Streptococcus pyogenes*	Erythromycin
	Macrolides
**GRAM-NEGATIVE**	
*Acinetobacter baumanii*	Carbapenems
	Imipenem
*Escherichia coli*	Ampicillin
	Cephalosporins
	Chloramphenicol Fluoroquinolones
	Nalidixic acid Rifampin
	Sulfamethoxazole Streptomycin Tetracycline
*Klebsiella pneumonia*	Carbapenems
	Imipenem
*Pseudomonas aeruginosa*	β-lactams
	Chloramphenicol Fluoroquinolones Macrolides
	Novobiocin Sulfonamides Tetracycline
	Trimethoprim
*Salmonella typii*	Amoxycilin Ampicillin Chloroamphenicol
	Fluoroquinolones
	Trimethoprim
*Shigella flexneri*	Ciprofloxacin
	Nalidixic acid
*Vibrio cholera*	Fluoroquinolones Tetracycline

*Different antibiotics toward which Gram-positive and Gram-negative bacteria have developed resistance*.

AgNPs have been used alone or in combination with antibiotics. Namasivayam et al. (2011) evaluated and reported the antibacterial activity of AgNPs against drug-resistant pathogenic bacteria *Bacillus subtilis, E. coli, E. faecalis, K. pneumonia, P. aeruginosa*, and *S. aureus* (Namasivayam et al., 2011). Nanda and Saravanan (2009) evaluated AgNPs for their antimicrobial activity against methicillin resistant *S. aureus* (MRSA), methicillin-resistant *Staphylococcus epidermidis* (MRSE), *S. pyogenes, S. typhi*, and *K. pneumoniae*. The observed antibacterial activity was maximum in case of MRSA, intermediate in MRSE and *S. pyogenes*, whereas the antibacterial activity seen against *S. typhi* and *K. pneumonia* was moderate. In order to further improve the AgNPs-based therapeutics, the use of AgNPs-antibiotic combination against drug-resistant pathogenic strains is recommended. AgNPs have displayed synergistic antimicrobial effect when used in combination with antibiotics (Fayaz et al., 2010). The synergistic effect of 19 antibiotics and the silver–water dispersion solution was studied by De' Souza et al. (2006). The silver–water dispersion solution is produced by an electro-colloidal process and the dispersion solution contains AgNPs clusters of 15 nm diameter. In the study, the antimicrobial activity of amoxicillin and clindamycin was evaluated against some MDR strains such as *E. coli, S. aureus, S. typhi, Shigella flexneri*, and *B. subtilis*. While the combination of silver–water dispersion with amoxicillin or clindamycin had an additive effect on *B. subtilis, S. aureus* 6538 P strain, *S. flexneri*, and *S. typhi*, on the contrary, the AgNPs dispersion solution in combination with amoxicillin displayed an antagonistic effect toward methicillin-resistant *S. aureus* strain (MRSA) (De' Souza et al., 2006). Shahverdi et al. (2007) studied the additive effect of AgNPs antibacterial effect against *E. coli* and *S. auerus* in presence of antibiotics such as amoxicillin, clindamycin, erythromycin, penicillin G and vancomycin. Fayaz et al. (2010) demonstrated synergistic effect of AgNPs against both Gram-positive and Gram-negative bacteria in combination with antibiotics. In case of Gram-negative bacterium *S. typhi*, the potency of ampicillin-mediated cell wall lysis increases when a combination of AgNPs and antibiotic is used (Rajawat and Qureshi, 2012). This suggests that AgNPs must be increasing the local concentration of antibiotics at the site of action and thus improves their potency. Besides potency against MDR and non-MDR bacterial strains, AgNPs also act as a potent, fast-acting anti-fungal agent against a wide range of fungal genera such as *Aspergillus, Candida, Fusarium, Phoma*, and *Trichoderma sp*. (Duran et al., 2007; Gajbhiye et al., 2009). AgNPs have also synergisitc fungicidal activity against the *Candida albicans, Fusarium semitectum, Phoma glomerata, Phoma herbarum*, and *Trichoderma* sp. in combination a commercial antifungal agent, fluconazole (Gajbhiye et al., 2009).

## Effects of nanoscale and physico-chemical properties on antimicrobial activity of AgNPs

Development or synthesis of metal derived nanomaterials for biomedical applications depends upon a number of physical, chemical, thermal, electrical, and optical properties. Some properties have more significance in medical application while other properties have relevance in industrial and environmental applications. Unlike their “macro” counterpart, nanoparticles demonstrate unique and significantly effective physico-chemical properties that make nanoparticles suitable for their intended use in improved healthcare. Several studies have demonstrated that bactericidal properties of the AgNPs are strongly influenced by their shape, size, concentration, and colloidal state (Pal et al., 2007; Bhattacharya and Mukherjee, 2008; Rai et al., 2012; Nateghi and Hajimirzababa, 2014; Raza et al., 2016). It has been found that reducing the size of AgNPs enhances their stability and biocompatibility (Kim et al., 2005, 2011). Hence, it is necessary to design appropriate sized, shaped nanoparticles with desirable surface properties for use in a diverse range of clinical and therapeutic interventions.

Shape of the nanoparticles is one of the properties, which affects other physico-chemical properties of the nanoparticles (Burda et al., 2005). AgNPs interacts with bacteria, fungi and viruses in a shape-dependent manner (Panacek et al., 2009; Galdiero et al., 2011; Tamayo et al., 2014; Wu et al., 2014; Raza et al., 2016). Energy-filtering TEM images have revealed alterations in the cell membrane of the gram negative *E. coli* bacterium upon treatment with differently shaped AgNPs, both in liquid and semi-solid agar medium (Pal et al., 2007). As compared to the spherical or rod-shaped AgNPs, truncated triangular shaped AgNPs show enhanced antibacterial action (Chen and Carroll, 2002; Pal et al., 2007). AgNPs with the same surface areas, however, different shapes show differential bactericidal activity, which can be attributed to the variations in the effective surface areas and active facets of AgNPs. Different surface chemistries, such as foamy carbon, poly (N-vinyl-2-pyrrolidone) (PVP), and bovine serum albumin (BSA) can also influence AgNPs interaction with viruses, such as HIV-1 virus, and causes their inhibition (Elechiguerra et al., 2005). Since, both BSA and PVP are completely encapsulated and are bounded directly to the nanoparticle surface, there is fundamentally no exposed surface area for AgNPs-virus interaction. On the contrary, the foamy carbon silver nanoparticles, which display an exposed surface area for virus attachment, display higher cytotoxicity and cause inhibition comparatively higher than AgNPs with BSA and PVP surface chemistry (Elechiguerra et al., 2005). However, there is limited information available about how shape of the nanoparticles influences AgNPs biological activity.

Another important physico-chemical property of AgNPs is their size. In general, for nanoparticles to be effective their size typically should be no larger than 50 nm. More precisely, silver nanoparticles with size between 10 and 15 nm have increased stability, biocompatibility and enhanced antimicrobial activity (Yacaman et al., 2001). Some studies have revealed that the antibacterial action of AgNPs is more effective against *S. aureus* and *K. pneumoniae* when nanoparticles of smaller diameter (<30nm) are used (Collins et al., 2010). The antibacterial effect of AgNPs as proposed is due to their smaller particles size that apparently has superior penetration ability into bacteria, especially in Gram-negative (Morones et al., 2005). AgNPs of 5–10 nm dimension display both bacteriostatic as well as bactericidal effects against *S. aureus*, MSSA and MRSA (Ansari et al., 2011). Espinosa-Cristobal et al. (2009) tested the potential of different sized AgNPs against *Streptococcus mutans*, a causal organism of dental caries, and suggested that as the AgNPs particle size diminishes, the antibacterial activity increases. Interestingly, the attachment of AgNPs with the cell membranes and resulting alterations in lipid bilayer lead to increased membrane permeability, damage and cell death, a potent antibacterial effect seemingly more pronounced when smaller sized nanoparticles are used (Li et al., 2013). To this end, Pal et al. (2007) demonstrated that the surface area to volume ratio of AgNPs and the crystallographic surface structures are important factors that determine the antibacterial activity of AgNPs.

AgNPs have been evaluated for their antiviral action mode against HIV-1 using a number of *in vitro* experiments, where at non-cytotoxic concentrations AgNPs exerted the antiviral activity against HIV-1; however, the mechanism underlying their HIV-inhibitory activity remained unclear (Sun et al., 2005). AgNPs are known to interact with HIV-1 virus via binding to gp120 glycoprotein knobs in a size-dependent manner (Elechiguerra et al., 2005). Nanoparticles usually of size between 1 and 10 nm attaches to the HIV-1 virus by binding to the disulfide bond regions of the CD4 domain present in the gp120 glycoprotein of the viral envelop (Elechiguerra et al., 2005). Other studies demonstrated that AgNPs ranging 5–20 nm diameter can inhibit replication of HIV-1 (Sun et al., 2005; Lu et al., 2008; Suganya et al., 2015). In this perspective, the size of the nanoparticles has substantial impact on antiviral potency of the AgNPs, which can be further enhanced by optimizing AgNPs size at nanolevel. Another case of size-dependent interaction of AgNPs with virus is AgNPs-Hepatitis B virus (HBV) interaction studied in a human hepatoma cell line, HepAD38 (Lu et al., 2008). Using UV-vis absorption titration assay, the *in vitro* binding affinity of different sized AgNPs (10–50 nm) for HBV DNA and extracellular virions was ascertained and the binding caused inhibition of HBV specific RNA and extracellular virions synthesis (Lu et al., 2008). In this regard, it is imperative to infer the significance of high binding of AgNPs for HBV DNA and its role in preventing virions from entering into the host cells. In this regard, *in vivo* studies with AgNPs are necessary for designing anti-viral vaccines with high beneficial therapeutic breakthroughs and low potential side effects.

The antibacterial effect is also concentration-dependent, but the effect is independent of acquisition of drug resistance by the bacteria. Ayala-Nunez et al. (2009) reported a dose-dependent antimicrobial activity of AgNPS against MRSA and non-MRSA and found that both MRSA and non-MRSA are discouraged in culture inoculums (conc. 10^5^-CFU per ml) at concentrations over 1.35 × 10^−3^ μg/ml. Studies on AgNPs antibacterial activity against Gram-positive *S. aureus* and Gram-negative *E. coli* have showed that the inhibition of the growth in case of *S. aureus* is less remarkable, while *E. coli* is inhibited at low AgNPs concentrations (Kim et al., 2007). Interestingly, the Gram-positive bacteria, such as *S. aureus, P. aeruginosa*, and *V. cholera*, are less susceptible than Gram-negative bacteria, such as *E. coli* and *S. typhi*; however, both classes of bacteria display complete growth inhibition at higher AgNPs concentrations (>75 μg/mL) (Kim et al., 2007).

**Figure 1:**
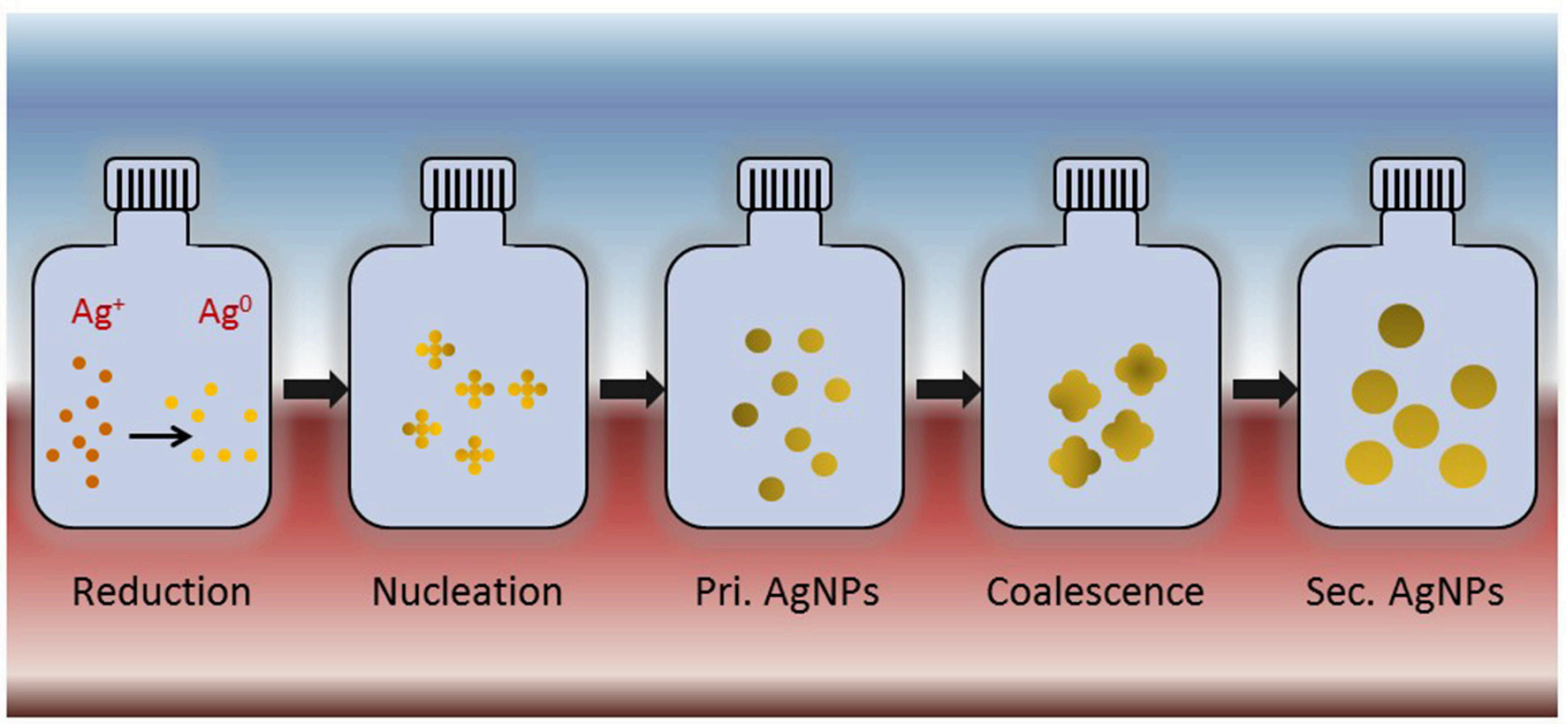
**Schematic representation of synthesis of colloidal silver nanoparticles using chemical reduction process**. Silver ions (Ag^+^) subjected to chemical reduction to form silver atoms (Ag^0^). These atoms undergo nucleation to form primary AgNPs that further coalesce with each other to form final AgNPs.

AgNPs in colloidal form, i.e., suspended nano-sized silver particles, have shown enhanced antimicrobial potential over AgNPs alone in a number of studies (Sondi and Salopek-Sondi, 2004; Panacek et al., 2006; Lkhagvajav et al., 2011). Colloidal AgNPs are synthesized using chemical reduction, physical, biological and green method using plant extract (Iravani, 2011; Iravani et al., 2014). The chemical reduction method of colloidal AgNPs synthesis is the most popular method (Figure 1). Colloidal state of AgNPs is an essential attribute for their antimicrobial activity. On the contrary, AgNPs in liquid system have showed only limited applications as bacteriocidal agents because of their low colloidal stability (Kumar et al., 2014; Shi et al., 2014). Colloidal silver appears to be a powerful, antibacterial therapy against infections because it serves as a catalyst and destabilize the enzymes that pathogenic drug-resistant bacteria, fungi, yeast, and viruses essentially need for their oxygen utilization (Dehnavi et al., 2012; Kumar et al., 2014; Suganya et al., 2015). For instance, colloidal AgNPs possess enhanced bactericidal potential against drug-resistant Gram-positive and Gram-negative bacteria and MRSA (Sondi and Salopek-Sondi, 2004; Panacek et al., 2006; Lkhagvajav et al., 2011). The enhanced bactericidal potential of the AgNPs has been correlated to their colloidal stability in the medium. Colloidal stability of AgNPs has also been suggested to regulate signal transduction pathways in bacteria by altering the phosphotyrosine profile of the proteins, which leads to growth inhibition in bacteria (Shrivastava et al., 2007). Colloidal AgNPs synthesized using sol-gel method with size 20–45 nm were found effective against bacterial strains such as *E. coli, S. aureus, B. subtilis, Salmonella typhimurium, P. aeruginosa*, and *K. pneumoniae* as well as fungal strain *C. albicans* at MIC of 2–4 μg/ml (Lkhagvajav et al., 2011). In addition, use of γ-radiation (Shin et al., 2004), microwave irradiation (Phong et al., 2009), Tollen's process (Yin et al., 2002) and rational use of mechanistic understanding (Wuithschick et al., 2013) has made it possible to produce colloidal AgNPs with smaller size and narrow size distribution. Recently, green synthesis has come up as a novel synthesis procedure for producing colloidal AgNPs with controlled size, high stability and improved antibacterial activity (Dehnavi et al., 2012).

### Mechanistic basis of antimicrobial activity of AgNPs

Antimicrobial efficacy of AgNPs was evaluated by many researchers against a broad range of microbes, including MDR and non-MDR strains of bacteria, fungi, and viruses. Nano-sized metal particles are now well-established as a promising alternate to antibiotic therapy because they possess unbelievable potential for solving the problem associated with the development of multidrug resistance in pathogenic microorganisms, hence also regarded as next-generation antibiotics (Rai et al., 2012). In particular, the use of AgNPs has gained much attention in this regard (Jana and Pal, 2007; Szmacinski et al., 2008; Stiufiuc et al., 2013). Although, AgNPs have been proved effective against over 650 microorganisms including bacteria (both Gram-positive and negative), fungi and viruses; however, the precise mechanism of their mode of antimicrobial action is not fully understood yet (Malarkodi et al., 2013).

**Figure 2:**
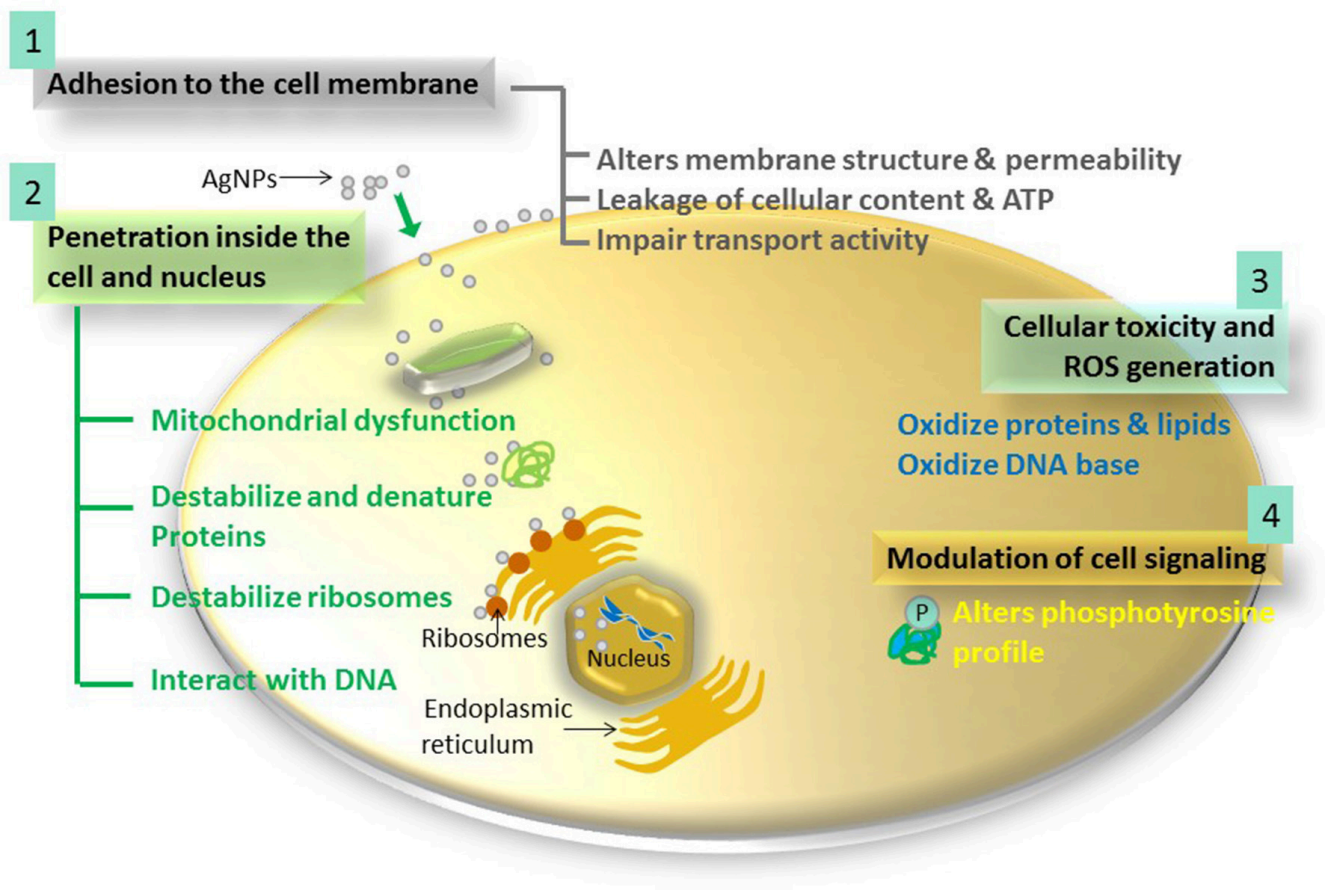
**The four most prominent routes of antimicrobial action of AgNPs**. 1, AgNPs adhere to microbial cell surface and results in membrane damage and altered transport activity; 2, AgNPs penetrate inside the microbial cells and interact with cellular organelles and biomolecules, and thereby, affect respective cellular machinery; 3, AgNPs cause increase in ROS inside the microbial cells leading to cell damage and; 4, AgNPs modulate cellular signal system ultimately causing cell death.

Nevertheless, some fundamental modes of antimicrobial action of AgNPs have been recognized (Figure 2, Table 2). Use of highly sophisticated techniques such as high resolution microscopic (AFM, FE-SEM, TEM, and XRD), spectroscopic (DLS, ESR spectroscopy, Fluorescence spectroscopy, Inductively coupled plasma-optical emission spectroscopy, UV-vis), molecular, and biochemical techniques have provided deep mechanistic insights about the mode of antimicrobial action of AgNPs (Sondi and Salopek-Sondi, 2004; Kim et al., 2007; Pal et al., 2007; Dehnavi et al., 2012; Rai et al., 2012). The antimicrobial action of AgNPs is linked with four well-defined mechanisms: (1) adhesion of AgNPs onto the surface of cell wall and membrane, (2) AgNPs penetration inside the cell and damaging of intracellular structures (mitochondria, vacuoles, ribosomes) and biomolecules (protein, lipids, and DNA), (3) AgNPs induced cellular toxicity and oxidative stress cause by generation of reactive oxygen species (ROS) and free radicals, and (4) Modulation of signal transduction pathways. Besides these four well-recognized mechanisms, AgNPs also modulate the immune system of the human cells by orchestrating inflammatory response, which further aid in inhibition of microorganisms (Tian et al., 2007).

**Table 2:** **Mode of antimicrobial action of AgNPs**.

**Bacterial Strain**	**AgNPs size (nm)**	**Mode of action**	**References**
**GRAM-POSITIVE**			
*Bacillus subtilis*	5	Cell membrane damage; leakage of reducing sugars	Li et al., 2013
	10	Degradation of chromosomal DNA; increase in ROS levels	Hsueh et al., 2015
*Clostridium diphtheria*	28.42	Rupture of the cell wall; denaturation of proteins	Nalwade and Jadhav, 2013
*Listeria monocytogenes*	–	Penetration inside the bacteria	Tamayo et al., 2014
	23 ± 2	Dysfunction of electron transport chain; increase in ROS levels at cell membrane	Belluco et al., 2016
*Staphylococcus aureus*	–	Adhesion to cell wall; cell membrane detachment from cell wall; DNA condensation; inhibition of replication; inactivation of proteins	Feng et al., 2000
	5	Cell membrane damage; leakage of reducing sugars	Li et al., 2013
	25	Interaction with cell membrane; interaction with S- and P-containing compounds; inhibition of respiration	Panacek et al., 2006
**GRAM-NEGATIVE**			
*Escherichia coli*	5 ± 2	Interaction with cell membrane; interaction with S- and P-containing compounds	Morones et al., 2005
	–	Adhesion to cell wall; cell membrane detachment from cell wall; DNA condensation; Inhibition of replication; inactivation of proteins	Feng et al., 2000
	10	Interaction with S- and P-containing compounds	Pal et al., 2007
	5	Cell membrane damage; leakage of reducing sugars	Li et al., 2013
	1–10	Interaction with cell membrane; increase in membrane permeability; improper transport activity; leakage of cellular components	Sondi and Salopek-Sondi, 2004
	25	Interaction with S- and P-containing compounds	Panacek et al., 2006
	16	Interaction with cell membrane; interaction with S- and P-containing compounds	Raffi et al., 2008
	–	Destabilization of ribosomes; inhibition of protein synthesis; inhibition of expression of enzymes required for ATP generation	Lok et al., 2006
	9.3	Interaction with cell membrane	Mirzajani et al., 2011
*Klebsiella pneumonia*	<50	Interaction with DNA; inhibition of cell division	Kumar et al., 2016
*Pseudomonas aeruginosa*	5 ± 2	Interaction with cell membrane; interaction with S- and P-containing compounds	Morones et al., 2005
	10	Penetration inside the cell	Habash et al., 2014
	28	Attenuation of quorum sensing	Singh et al., 2015
*Salmonella typii*	5 ± 2	Interaction with cell membrane; interaction with S- and P-containing compounds	Morones et al., 2005
	2–23	Cell wall lysis	Rajawat and Qureshi, 2012
*Vibrio cholera*	5 ± 2	Interaction with cell membrane; interaction with S- and P-containing compounds	Morones et al., 2005
	90–100	Inhibition of metabolic pathways	Salem et al., 2015

*Cellular targets and antibacterial activity of AgNPs against different multidrug-resistant Gram-positive and Gram-negative strains*.

### Adhesion of AgNPs onto the surface of cell wall and membrane

AgNPs exposure to microorganisms causes adhesion of nanoparticles onto the cell wall and the membrane. The positive surface charge of the AgNPs is crucial for the adhesion (Abbaszadegan et al., 2015). The positive charge confers electrostatic attraction between AgNPs and negatively charged cell membrane of the microorganisms, thereby facilitates AgNPs attachment onto cell membranes. Morphological changes become evident upon such interaction and can be characterized by shrinkage of the cytoplasm and membrane detachment finally leading to rupture of cell wall (Nalwade and Jadhav, 2013). Transmission electron microscopy has revealed that after a few minutes of contact with AgNPs, the cell membrane of *E. coli* cells gets completely disrupted (Raffi et al., 2008). The cell wall becomes circumferential and numerous electron dense pits can be seen at sites of damages induced by AgNPs, as visualized by TEM (Sondi and Salopek-Sondi, 2004). Besides electrostatic attraction, the interaction of AgNPs with the sulfur-containing proteins present in the cell wall causes irreversibly changes in cell wall structure resulting in its disruption (Ghosh et al., 2012). This in turn affects the integrity of lipid bilayer and permeability of the cell membrane. The alterations in cell morphology cause increase in membrane permeability, which affects cells ability to properly regulate transport activity through the plasma membrane (Schreurs and Rosenberg, 1982). For instance, silver impairs the uptake and release of phosphate ions in *E. coli* (Schreurs and Rosenberg, 1982). Similarly, silver ions can also alter transport and the release of potassium (K+) ions from the microbial cells. Besides affecting the transport activity, the increase in membrane permeability may have more pronounced effects such as loss by leakage of cellular contents, including ions, proteins, reducing sugars and sometimes cellular energy reservoir, ATP (Lok et al., 2006; Kim et al., 2011; Li et al., 2013). In fact, the proteomic data on AgNPs treated microbial cells have shown an accumulation of immature membrane precursor proteins that cause destabilization of the outer membrane of *E. coli* (Amro et al., 2000; Mirzajani et al., 2011). The translocation of precursor protein to the cell membrane require energy from proton motive forces and ATP, therefore, accumulation of immature precursor proteins suggests dissipation of proton motive forces and depletion of cellular ATP, the latter perhaps is due to leakage or inhibition of ATP synthesis (Yamanaka et al., 2005; Lok et al., 2006; Kim et al., 2007, 2011; Raffi et al., 2008; Mirzajani et al., 2011). Microbial cells exposed to AgNPs also suffer genetic alterations such as condensation of genetic material (Feng et al., 2000; Sondi and Salopek-Sondi, 2004; Kim et al., 2011). As a consequence, several vital cellular functions get inhibited that ultimately lead to cell necrosis and death (Rai et al., 2012, 2014).

Additionally, the antimicrobial potential of AgNPs is also influenced by the thickness and composition of the cell wall of the microorganisms. Gram-negative bacteria, such as *E. coli*, are more susceptible to AgNPs than Gram-positive bacteria, such as *S. aureus*. This is due to difference in the organization of a key component of the cell membrane, peptidoglycan. In Gram-positive bacteria, the cell wall is composed of negatively charged peptidoglycan layer (30 nm thickness) and the amount of peptidoglycan is comparatively more in Gram-positive than Gram-negative bacteria (~3–4 nm thickness). In nutshell, the less liability of Gram-positive bacteria to antibiotic therapy can be explained on the basis of the fact that their cell wall is comparatively much thicker than that of Gram-negative bacteria (Rai et al., 2012). The thicker cell wall of Gram-positive as well as the negatively charge of the peptidoglycan leave silver ions stuck onto the cell wall. For this reason, *S. aureus*, a Gram-positive bacterium, which possesses a thick cell wall and more peptidoglycan molecules, prevents the action of the silver ions and renders bacterium comparatively more resistant to antimicrobial therapy of the AgNPs (Feng et al., 2000). In contrast, Gram-negative bacteria are more susceptible to AgNPs-based antimicrobial therapy owing to less thicker cell wall and less peptidoglycan (Pal et al., 2007). In addition, Gram-negative bacteria contain lipopolysaccharides (LPS) in the cell membrane, which contributes to structural integrity of the membrane as well as protect the membrane from chemical attacks. However, the negative charge of LPS promotes adhesion of AgNPs and makes bacteria more susceptible to antimicrobial therapy. Several studies have shown the pronounced adhesion and deposition of AgNPs onto the cell surface, in particular, of the Gram-negative bacteria due to the presence of LPS in their cell membrane (Pal et al., 2007). These differences in structure, thickness and composition of cell can explain why Gram-positive *S. aureus* are less inhibited and Gram-negative *E. coli* shows substantial inhibition even at low antibiotic concentrations (Kim et al., 2007). In this perspective, it can be ruled out that there exists a correlation between the concentration (effective dose) of the AgNPs and the class of the bacteria treated owing to differences in the cell wall structure, thickness and composition.

### AgNPs penetration inside the cell and destabilization of intracellular structures and biomolecules

There are several cellular dysfunctions that result from interaction of AgNPs with the microbial cell membrane. In first instance, AgNPs only attach to cell membrane and alter membrane structure, permeability and transport activity. In other case, after adhesion to the cell membrane, the AgNPs may also penetrate inside the cells and affect vital cellular functioning (Habash et al., 2014; Singh et al., 2015). Transmission electron microscopic (TEM) images have depicted AgNPs inside the *E. coli* cells (Lok et al., 2006). Kvitek et al. (2008) suggested that the use of surfactants and anionic detergents such as sodium dodecyl sulfate (SDS) greatly enhance the antimicrobial activity of the AgNPs. Porins, water-filled channels present in outer membrane (OM) of the Gram-negative bacteria, are involved in the uptake of AgNPs inside the bacterial cells. As expected, the expression of mutated porin proteins in *E. coli* rendered cells more resistant to AgNPs based antibacterial action therapy (Li et al., 1997). In situation when AgNPs penetrate inside the microbial cell, it may interact with cellular structures and biomolecules such as proteins, lipids, and DNA. Interactions with cellular structures and biomolecules have respective damaging effects on microbes. In particular, AgNPs interaction with ribosomes leads to their denaturation causing inhibition of translation and protein synthesis (Morones et al., 2005; Jung et al., 2008; Rai et al., 2012). It has been shown that Ag (+) ions may interact with the functional groups of the proteins, resulting in their deactivation. For instance, Ag (+) ions bind to thiol groups (ASH) of the protein present in the cell membrane forming stable SAAg bonds resulting in protein deactivation (Klueh et al., 2000; Rai et al., 2012). The proteins are involved in transmembrane ATP generation and mediating ion transport across cell membrane (Klueh et al., 2000). Both AgNPs and Ag (+) ions alter the 3D structure of proteins upon interaction and interfere with disulfide bonds and block active binding sites leading to overall functional defects in the microorganism (Lok et al., 2006). Steuber et al. (1997) described a mechanism for Ag(+) antibacterial action in Gram-negative *Vibrio alginolyticus* in which FAD gets displaced from the holo-enzyme Na+-NQR resulting in complete loss of enzyme activity. Bactericidal effect of AgNPs has also been linked with blocking of sugar metabolism. Bhattacharya and Mukherjee (2008) demonstrated that inhibition of sugar metabolism is due to inactivation of the enzyme phosphomannose isomerase upon interaction with AgNPs. Phosphomannose isomerase mediates the isomerization of mannose-6-phosphate into fructose-6-phosphate, the latter is an important intermediate in the glycolytic cycle. Additionally, interaction of AgNPs with DNA may cause shearing or denaturation of the DNA and interruption in cell division (Hsueh et al., 2015; Kumar et al., 2016). AgNPs causes DNA damage (such as strand breaks) and mutations in essential DNA repair genes (mutY, mutS, mutM, mutT, and nth) in *E. coli* making mutant strains, rather than wild type, more susceptible to AgNPs based antibacterial therapy (Radzig et al., 2013). It has been found that Ag (+) ions form complex with nucleic acids, where it preferentially interact with the nucleosides but not with the phosphate (${\text{PO}}_{4}^{-}$) group of the nucleic acids. Ag (+) ion intercalates between the purine and pyrimidine base pairs, disrupts the H-bonds between base pairs of the anti-parallel DNA strands, and thereby, disrupts the double helical structure (Klueh et al., 2000). Intercalation of AgNPs in the DNA helix may block the transcription of genes in microorganisms (Morones et al., 2005). AgNPs also causes DNA molecule to change its state from relaxed to condensed form, the latter results in loss of replication ability (Feng et al., 2000). When AgNPs interacts with *S. aureus*, the cell division is inhibited in its initial stages (Jung et al., 2008) suggesting that the interaction of the Ag (+) ions with DNA may have role in prevention of cell division and reproduction (Monteiro et al., 2012).

### AgNPs induced cellular toxicity and oxidative stress

Increase in cellular oxidative stress in microbes is an indication of toxic effects caused by heavy metals ions, such as Ag (+).Therefore, increased concentration of Ag (+) ions is expected to cause an increase in cellular oxidative stress. The potent antibacterial, antifungal and antiviral activity of AgNPs is due to their ability of producing ROS and free radical species such as hydrogen peroxide (H_2_O_2_), superoxide anion (O2^−^), hydroxyl radical (OH•), hypochlorous acid (HOCl) and singlet oxygen (Kim et al., 2011). The antibacterial potential of AgNPs is related with the generation of free radicals and reactive oxygen species (ROS) and consequent increase in oxidative stress in cells (Pellieux et al., 2000; Kim et al., 2005, 2007; Wu et al., 2014). Reactive oxygen species are also generated intracellularly during mitochondrial oxidative phosphorylation. Molecular oxygen generates O_2_•, the primary ROS, via one-electron reduction catalyzed by nicotinamide adenine dinucleotide phosphate (NADPH) oxidase. Further reduction of molecular oxygen may takes place via dismutation and metal-catalyzed Fenton reaction, forming either H_2_O_2_ or OH•, respectively (Vallyathan and Shi, 1997; Thannickal and Fanburg, 2000). The generation of ROS in bacterial cells causes cell death, although, the precise mechanism of ROS-mediated antibacterial activity of AgNPs is not fully clear (Pellieux et al., 2000). This toxic effect may be due to the binding of Ag (+) ions onto the cell membrane of the microbes, which consequently relay signaling and blocks the mitochondrial respiratory function of the microbes (Blecher and Friedman, 2012). Ag (+) ions are known cause dysfunction of respiratory electron transport chain by uncoupling it from oxidative phosphorylation by inhibiting respiratory chain enzymes (Belluco et al., 2016). Excessive amount of generated free radical causes direct damage to mitochondrial membrane causing necrosis, and eventually, cell death. Other outcomes of increase in ROS levels in the cells include hyperoxidation of lipids, proteins and DNA (Huang et al., 2010). Free radicals also interact with lipids, abundantly present in biomembranes, to yield lipid peroxidation products having implications in mutagenesis. Polyunsaturated fatty acids are subject to oxidation giving rise to lipid hydroperoxides as the initial step in ROS generation (Howden and Faux, 1996). Prooxidant metals such as Cu and Fe react with these lipid hydroperoxides to induce DNA damaging end-products malondialdehyde (MDA) and 4-hydroxynonenal that act as inflammatory mediators and risk factors for carcinogenesis (Howden and Faux, 1996). AgNPs triggered free radicals cause reduction of glutathione (GSH) into its oxidized form glutathione disulfide (GSSG), thereby contributing to oxidative stress, apoptosis, and activation to oxidative signaling pathways (Rahman, 2007; Fenoglio et al., 2008). NP-mediate ROS generation also modulate the antioxidant activities of ROS-metabolizing enzymes such as NADPH-dependent flavoenzyme, catalase, glutathione peroxidase, and superoxide dismutase (Stambe et al., 2004).

Increase ROS levels caused due to mitochondrial stress, ER stress, and deactivation of anti-oxidant enzymes in the cells consequently promote genotoxic effects. Nanoparticles induced genotoxicity includes chromosomal aberrations such as mutations, DNA strand breaks, and oxidative DNA base damage (Xie et al., 2011). Hydroxyl radical (OH•), one of the highly potent radicals, and is known to react with all components of DNA causing DNA single strand breakage via formation of 8-hydroxyl-2′-deoxyguanosine (8-OHdG) DNA adduct (Pilger and Rüdiger, 2006; Valavanidis et al., 2009). 8-OHdG is a biomarker of OH -mediated DNA lesions. Taken all together, the available data suggest that the most drastic antimicrobial effects are associated with AgNPs-induced ROS generation and increase in oxidative stress having both cytotoxic as well as genotoxic effects.

### Modulation of signal transduction pathways

Phosphorylation of various protein substrates in bacteria is widely recognized (Deutscher and Saier, 2005). The cycle of phosphorylation and dephosphorylation cascade is mechanism of signal relay in microorganisms essential for microbial growth and cellular activity (Kirstein and Turgay, 2005). Examining the phosphotyrosine profile of bacterial proteins from both Gram-positive and Gram-negative offers a useful way to study the effect of AgNPs on bacterial signal transduction pathways. These signaling pathways affect bacterial growth and other molecular and cellular activities. The reversible phosphorylation of tyrosine residue of protein substrates such as RNA polymerase sigma factor (RNA pol σ factor), single-stranded DNA binding proteins (ssDBPs) and UDP glucose dehydrogenase lead to their activation (Mijakovic et al., 2003, 2005, 2006). The resultant phosphorylated proteins have essential role in DNA replication, recombination, metabolism and bacterial cell cycle. Therefore, inhibition of phosphorylation of proteins would inhibit their enzymatic activity, which in turn will result in inhibition of bacterial growth. Similarly, Iniesta et al. (2006) also suggested phosphosignaling pathways to be critical for progression of cell cycle in bacteria. In addition, tyrosine phosphorylation of protein has also been implicated in the biosynthesis and transport of exopolysaccharide and capsular polysaccharide in a number of Gram-positive and Gram-negative bacteria (Grangeasse et al., 2003). AgNPs putatively modulates cellular signaling and acts by dephosphorylating tyrosine residues on key bacterial peptide substrates and thus inhibit microbial growth (Shrivastava, 2008). Treatment of *S. aureus* with AgNPs have showed no change in the profile of tyrosine phosphorylated proteins; however, treatment of *E. coli* and *S. typhi* with AgNPs has resulted into noticeable change in dephosphorylation of two peptides of relative masses 150 and 110 kDa (Shrivastava et al., 2007). It is necessary to conduct biochemical characterization of the two peptide substrates dephosphorylated in *E. coli* and *S. typhi* and to identify the putative tyrosine phosphatases that cause their dephosphorylation as described by Shrivastava et al. (2007).

## AgNPs exposure to human cells and tissues: implications of cytotoxicity, genotoxicity and inflammatory response in diseases

**Figure 3:**
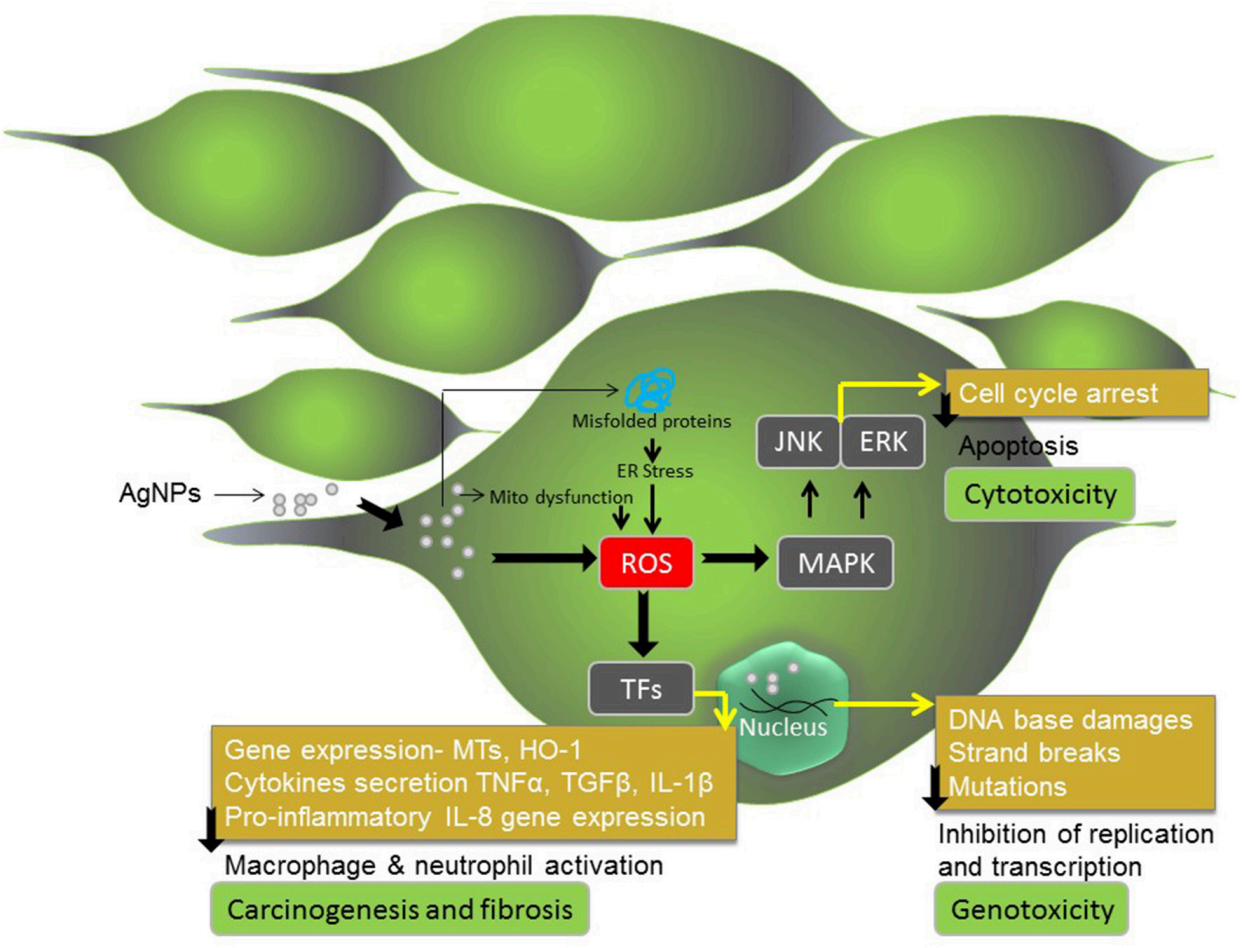
**AgNPs exposure to human or mammalian cells**. AgNPs induce cytotoxic, genotoxic and inflammatory response in human and mammalian cells and consequently trigger apoptotic cell death, carcinogenesis and fibrosis.

The most critical and fundamental problem associated with the use of Silver nanoparticles or any other nanoparticles in human disease treatment, therapeutic intervention and drug delivery is their biosafety and biocompatibility aspect. Increasing applications of AgNPs based antimicrobial therapies are raising concerns regarding the biosafety and clinical risks associated with them to human. Our body's immune system confers a state of protection from foreign invaders, first by discriminating the “self” and “non-self” antigens and second by recruiting a dynamic network of immune cells in a coordinated fashion to neutralize the “non-self” antigens. AgNPs are recognized as “non-self,” and therefore, AgNPs evokes immune response. AgNPs modulates both the cellular and humoral immune response system. Several studies conducted on different human cells explained and extended our understanding about the underlying molecular mechanism of AgNPs induced cytotoxicity, genotoxicity and inflammatory response related to fibrosis and carcinogenesis (Valko et al., 2006; Asharani et al., 2009a; Kennedy et al., 2009; Manke et al., 2013). In context to cells exposed to AgNPs, increase in cytotoxicity, genotoxicity, and activation of signaling and inflammatory response, to a large extend, is associated with generation of ROS, free radicals and consequently buildup of oxidative stress (Ahamed et al., 2010; Johnston et al., 2010; Figure 3). Acting as signal molecules, ROS and free radicals can cause hyperoxidation of cell organelles and disruption of mitochondrial activity. In this regard, TEM image analysis has confirmed that AgNPs interact with mitochondria (Menu et al., 2012) and this interaction affect mitochondrial respiration chain resulting in mitochondrial stress. Similarly, AgNPs interacts with proteins and unfolds or misfolds them leading to unfolded protein response (UPR) and endoplasmic reticulum (ER) stress (Asharani et al., 2009b; Zhang et al., 2012). Both mitochondrial stress and ER stress have additive effect on ROS generation in cell and apoptotic cell death, generally referred to as cytotoxicity (Asharani et al., 2009a; Menu et al., 2012). Asharani et al. (2009b) also demonstrated that AgNPs penetrates inside the nucleus based on TEM microphotographs (Menu et al., 2012). AgNPs inside nucleus induce 8-Oxoguanine (8-oxoG) oxidative base damages, strand-breaks and mutations in DNA leading to so called genotoxicity (Ahamed et al., 2008; Foldbjerg et al., 2009; Kim et al., 2009; Hudecová et al., 2012). AgNPs can also mediate oxidative-sensitive activation of signaling cascades and inflammatory response leading to fibrosis and carcinogenesis (Manke et al., 2013).

The AgNPs-induced toxicity depends on nanoparticles size, concentration and duration of the treatment (Ahamed et al., 2010; Johnston et al., 2010). Upon exposure to AgNPs, human cells and tissues experience increased levels of oxidative stress and in order to counteract the overwhelming oxidative stress, cells respond via diverse mechanisms (Huang et al., 2010). We referred oxidative stress to be mild, intermediate or high based on the concentration of AgNPs used during treatment.

Treatment of human and mammalian cells at AgNPs concentration below 10 μg/mL for 24 h causes mild oxidative stress condition. This concentration of AgNPs usually causes a decrease in cell viability by ~30% (Çiftçi et al., 2013; Kreeinthong and Uawithya, 2014). At this concentration, the cells once treated with AgNPs may recover growth if transferred to fresh medium devoid of AgNPs. AgNPs have been shown to produce intracellular ROS even at the lower concentration of 1 μg/mL. The ROS and superoxide (O2•−) anions starts producing by the cells just after 10 min of exposure and shows maximal levels at 30 min and thereafter ceased to produce. Similarly, even at the low concentration of AgNPs (0.5 and 1 μg/ml), the condensation of genetic material become noticeable in human lymphoma cells (Eom and Choi, 2010). This suggests that, in order to counteract mild oxidative stress, cells after 30 min post-treatment mainly respond via cellular antioxidant enzyme systems. The nuclear factor (erythroid-derived 2)-like 2 (Nrf2) may be involved in induction of transcriptional activation of antioxidant genes (Manke et al., 2013).

During condition of the intermediate oxidative stress (10–40 μg/mL AgNPs), cells respond via up-regulation and down-regulation of a number of cellular pathways, including expression of gene coding for antioxidant proteins (Xu et al., 2012). The intermediate levels of oxidative stress result in activation of cellular signaling cascades, transcription factors (TFs), and cytokine so as to mount diverse range of cellular responses to combat stress induced damages. In particular, redox-sensitive mitogen-activated protein kinase (MAPK) pathway, and transcription factors such as AP-1, Nrf-2, and nuclear factor kappa-B (NF-κB) are known to play important role (Lee et al., 2009; Manke et al., 2013). At intermediate oxidative stress level, cells viability reduces below 50% upon treatment with 40 μg/mL AgNPs (Çiftçi et al., 2013). Based on experimental data, at this concentration cells show maximum apoptotic effect (Çiftçi et al., 2013), which may be attributed to both AgNPs-induce cytotoxicity (mitochondrial dysfunction) and AgNPs-mediated DNA damage and genotoxicity (cell cycle arrest in G2/M phase) (Asharani et al., 2009a). In fact, *in vitro* studies conducted on a number of other cell lines such as pulmonary fibroblasts, epithelial cells, melanoma cells, and hepatoma cells showed that AgNPs at a concentration of 13.45 μg/mL results in significant increase in membrane leakage of LDH and reduction in mitochondrial function (Ávalos Fúnez et al., 2013). Similarly, treatment of human-derived keratinocyte cell line (HaCaT) with 11–36 μg/mL AgNPs caused a reduction in mitochondrial function (Zanette et al., 2011).

Rinna et al. (2015) evaluated the role of AgNPs-generated ROS, as mediator of activation of MAPK pathways, in particular the three best-studied pathways: p38, ERK and JNK. The study showed that AgNPs-generated ROS caused phosphorylation of ERK and JNK but not p38. The study also revealed that the ROS induces oxidative DNA damage directly without involvement through the activation of MAPK pathways. However, the role of the ERK pathway was found to be crucial in the repair of oxidative DNA damages and inhibition of MEK, an upstream component of ERK, completely abolished the repair process. Consistent with this, the activation of ERK as well as of JNK was highest when the levels of ROS as well as oxidative damage to the DNA were recorded maximum (Rinna et al., 2015). This suggests that ERK pathway plays an important role in counteracting genotoxicity induced by AgNPs. Whereas, Eom and Choi (2010) suggested involvement of p38 MAPK activation in DNA damage, and consequently inhibition of cell cycle progression and apoptosis as mechanisms for both cytotoxicity and genotoxicity in Jurkat T cells by AgNPs. AgNPs have been found to act through ROS and JNK for induction of apoptosis in NIH3T3 cells (Hsin et al., 2008). In contrast, Asharani et al. (2009a) showed the direct role of AgNPs in the dysfunctioning of mitochondrial and apoptotic death in lung fibroblast IMR-90 cells. This suggests that JNK pathway plays an important role in counteracting cytotoxicity induced by AgNPs, but the effect is cell line dependent. There is possible involvement of protein acting downstream to ERK in modulation of cell cycle and apoptosis (Zhang and Liu, 2002; Mebratu and Tesfaigzi, 2009). However, further studies are necessary to elucidate and define the role of ERK downstream protein components in signaling cascades that may have role cell survival and apoptosis after AgNPs treatment.

The global transcriptional analysis of genes revealed that AgNPs treatment (Conc 15.2 μg/mL) to HeLa cells for 24 or 48 h causes differential expression of thousands of genes (Xu et al., 2012). Interestingly, number of genes that up-regulate or down-regulate after AgNPs treatment are comparatively more at 24 h than at 48 h of post-treatment. However, only a limited number of genes that up-regulate or down-regulate at 24 h post-treatment with AgNPs continue to express until the 48 h. The data suggest that the AgNPs-induced changes in gene expression are more pronounced upto 24 h post-exposure. Consistent with this, Rinna et al. (2015) also reported similar findings, where AgNPs induced concentration-dependent (1–25 μg/ml) increase in phosphorylation and activation of ERK1/2 and JNK1/2 MAP kinase signaling pathway (not p38 MAPK) after 30 min and upto 1 h. However, phosphorylated ERK1/2 and JNK1/2 decreased to levels almost same to the control levels after 1 h of post-treatment. Gene ontology and pathway analysis of the differentially regulated genes revealed that up-regulation of genes affects 14 signaling pathways; whereas down-regulation of genes influences 3 cellular pathways. The upregulated genes mainly entail changes of the 14 functional signal pathways closely associated with cell communication, cell-cell signaling, cell adhesion, signal transduction, intracellular signaling JAK-STAT cascade, metabolic processes, carbohydrate metabolic processes, lipid metabolic processes, response to stimulus, transport, endocytosis, cellular defense response, and immune response (Xu et al., 2012). On the contrary, the down-regulated genes mainly affect functional pathways such as the nucleic acid metabolic processes, cell cycle and mitosis (Xu et al., 2012). The presented results provide further evidences in support of AgNPs-induced activation and involvement of JAK-STAT pathway in mitochondrial dysfunction (cytotoxicity) and ERK1/2 and JNK1/2 (but not p38) in DNA damage induced cell cycle arrest (genotoxicity). Another reason for DNA damage induced cell cycle arrest (genotoxicity) could be interaction of AgNPs with actin cytoskeleton as suggested by Asharani et al. (2009a) and down-regulation of CDC14A as suggested by Xu et al. (2012), both at intermediate oxidative stress condition (AgNPs upto 40 μg/mL).

Under intermediate oxidative stress condition, the most prominent genes that upregulate upon AgNPs exposure are metallothionein genes and oxidative stress responsive genes that protect cells by neutralizing oxidative stress (Xu et al., 2012). Exposure of HeLa cells AgNPs results in over-expression some metallothionein isoforms such as MT1A, MT1F, MT1G, MT1X, and MT2A (Xu et al., 2011). Asharani et al. (2012) reported over-expression of the MT1F and HO-1 in IMR-90 cells upon AgNPs treatment. Kawata et al. (2009) also reported significant over-expression of three metallothionein genes, MT1H, MT1X, MT2A in human hepatoma cell line (HepG2 cells) after exposure to AgNPs. Recently, Xu et al. (2012) using microarray analysis demonstrated ten metallothionein genes (MT1F, MT1A, MT2A, MT1B, MT1G, MT1H, MT1X, MT1L, MT1M, and MT1E), heme oxygenase-1 (HO-1) and oxidative stress induced growth inhibitor 1 (OSGIN1) to be significantly up-regulated at 48 h of AgNPs-hydrogel treatment (Xu et al., 2012). To this end, metallothioneins (MTs) can be regarded as cellular biomarkers for AgNPs-induced cytotoxicity because these proteins act by facilitating metal detoxification and conferring protection from oxidative damages (Ruttkay-Nedecky et al., 2013).

The AgNPs concentration above the range of 40–50 μg/mL, depending upon cell type, causes extremely high oxidative stress condition and results in permanent damage to cells. The high oxidative stress condition caused by 50 μg/mL AgNPs or more in RAW264.7 cells and MCF-7 showed 70% reduction in cell viability (Çiftçi et al., 2013; Paul et al., 2015). Çiftçi et al. (2013) also found that at high AgNPs concentration, the apoptotic affect reduces, while the necrotic effect becomes prominent. This effect can be attributed oxidative stress induced damages to mitochondrial membrane and dysfunction of electron chain dysfunction, which ultimately lead to necrotic cell death.

To this end, increase cytotoxicity, genotoxicity, and inflammatory responses have implications in a number of diseases and disorders. NPs have been shown to up-regulate the expression of proinflammatory chemokine gene interleukin-8 (CXCL-8) via ROS, and NF-κB activation (Lee et al., 2009; Manke et al., 2013). Increase IL-8 secretion by the cells causes activation and recruitment of macrophages and neutrophils that increase the susceptibility of cancer progression and metastasis. Thus, ROS-NFκB mediated IL-8 expression response appears to be closely associated to factors driving cancer progression and metastasis (Genestra, 2007). The inflammatory cascade that involves profibrotic mediators such as TNF-α, IL-1β, and TGF-β have been implicated in the pathogenesis of fibrosis (Li et al., 2010).

## Conclusion

For centuries, silver-based compounds have been in use as an antimicrobial agent to discourage bacterial growth. There are already products in the market. For instance, AgNPs in Silver-Acticoat^TM^ dressing used for healing of chronic wounds and it has superior property than silver nitrate and silver sulphadiazine, the latter is a formulation of silver and sulphadiazine as 1% water-soluble cream (AgSD) (Dunn and Edwards-Jones, 2004; Pasupuleti et al., 2013). Polyvinyl alcohol (PVA) nanofibres impregnated with silver nanoparticles exhibit improved antibacterial potential against *E. coli* and *S. aureus*, and are considered suitable in wound dressings (Jun et al., 2007). Besides this, the susceptibility of inflammatory response in AgNPs based non-crystalline wound dressing therapy is remarkably much less (Fong and Wood, 2006). Other biomedical applications of AgNPs include impregnation of catheters and cardiovascular and bone implants with AgNPs for inhibiting biofilm formation and minimizing the chances of pathogenic growth (Tran et al., 2013). In orthopedics, AgNPs are loaded along with polymethyl methacrylate (PMMA) to be used as bone cementing material in synthetic joint replacement therapy (Alt et al., 2004). Out of all the metals (gold, platinum, Cu, Zn, Ti etc.) with antimicrobial properties, silver is known to exert the most effective antibacterial action. Nevertheless, Chen and Schluesener (2008) demonstrated that silver is comparatively non-toxic and non-mutageneic to human primary organ systems. Since, silver is non-cytotoxic to animal cells, AgNPs has been considered as a safe and promising antibactericidal agent against highly infectious drug-resistant bacteria such as *E. coli, P. aeruginosa*, and *S. aureus* (Namasivayam et al., 2011; El-Kheshen and El-Rab, 2012). AgNPs increases the antibacterial activities of antibiotics such as amoxicillin, clindamycin, erythromycin, penicillin, and vancomycin. However, similar to antibiotics, prolonged exposure of bacteria to AgNPs may result in the development of resistant bacterial cells. For instance, *E. coli* K12 MG1655 strain has developed resistance toward AgNPs; however, the bacterium does not possess any Ag-resistance element (Graves et al., 2015). It is therefore necessary in future to carefully examining the development of Ag-resistance in bacteria.

For synthesis of nanomaterials an array of physical, chemical and biological methods are available. A specific procedure is generally used to synthesize metal nanoparticles of desired property and performance. Silver nanoparticles can be easily synthesized using various procedures that include physical, chemical, electro-chemical and biological (Sun et al., 1989; Naik et al., 2002; Yin et al., 2003; Iravani et al., 2014). Certain other methods such as ultraviolet irradiation, laser ablation, photochemical reduction and some other have also been employed successfully, but are much expensive and require the use of harmful compounds. Therefore, there is an increasing demand for development cost-effective and environment-friendly methods. Advancement of this route (green synthesis) over chemical and physical approaches holds great promise. Green synthesis of nanoparticles is a cheap, eco-friendly, easily scaled up method for synthesis of nanoparticles that does not require use of high energy, pressure, temperature and toxic chemicals (Iravani, 2011). By optimizing and manipulating the synthesis procedures and using appropriate reducing agents and stabilizers, a specific control over shape, size, and charge distribution of the AgNPs can be achieved (Pal et al., 2007; Iravani, 2011; El-Kheshen and El-Rab, 2012).

AgNPs-associated cytotoxicity, genotoxicity and inflammatory response in cells have raised concerns of their inadvertent exposure in humans (Chopra, 2007). However, the cytotoxic, genotoxic, apoptotic and anti-proliferative effect of AgNPs can be used as a strategy against cancerous cells, such as cancer treatment of GBM patients (Gopinath et al., 2008; Urbańska et al., 2015). In this perspective, to encourage the safe use of AgNPs in disease therapy, long-term cytotoxicity, mutagenicity and carcinogenicity studies should be conducted in order to verify any adverse effects that may occurs during their use in therapeutics and drug delivery (Becker et al., 2011; Maneewattanapinyo et al., 2011; Klien and Godnic-Cvar, 2012; Tran et al., 2013). Some authors have conducted clinical trials using commercially available Silver nanoparticles (Munger et al., 2014, 2015; Smock et al., 2014). The trials are registered with Clinical-Trials.gov. These studies detected no clinically important alterations in metabolic, hematologic, or urinalysis profiles in humans. Besides this, no morphological changes were detected in the vital human organs such as lungs, heart or abdomen as well as no changes were recorded in pulmonary ROS formation or pro-inflammatory cytokine production (Munger et al., 2014).

In order to apply Silver nanoparticles based medicines for human therapeutic interventions and disease treatment, clinical trials must be conducted. However, there are some major roadblocks. In this perspective, we envisage that future studies must be conducted at three major levels before proposing AgNPs use in clinical trials, therapeutics interventions and drug delivery applications. First to synthesize AgNPs with unique physico-chemical properties using novel biofabrication procedures and techniques, second to verify if the microorganisms develop resistance toward the AgNPs based antimicrobial therapy, third to examine the cytotoxicity, genotoxicity, and inflammatory response of the AgNPs toward human cells. Although, there are some concerns and controversies related to AgNPs safe use in human diseases treatment and health care, the research done so far has suggested that AgNPs can be engineered to enhance its antimicrobial efficacy, stability, specificity, biosafety and biocompatibility for increased therapeutic benefits and reduced potential side effects.

## Author contributions

TD conceived the idea. TD, AK, and VY wrote the manuscript. TD prepared the tables and figures for the manuscript. TD, VY, and RM performed language editing. All authors read, reviewed and approved the manuscript.

### Conflict of interest statement

The authors declare that the research was conducted in the absence of any commercial or financial relationships that could be construed as a potential conflict of interest.
